# NEUROLOGICAL CORRELATES OF SOCIAL BONDING AND BRIDGING

**DOI:** 10.1093/geroni/igac059.951

**Published:** 2022-12-20

**Authors:** Mohit Manchella, Paige Logan, Brea Perry, Siyun Peng, Lucas Hamilton, Shannon Risacher, Andrew Saykin, Liana Apostolova

**Affiliations:** University of Southern Indiana, Carmel, Indiana, United States; Indiana University School of Medicine, Indianapolis, Indiana, United States; Indiana University, Bloomington, Indiana, United States; Indiana University, Bloomington, Indiana, United States; Indiana University, Bloomington, Indiana, United States; Indiana University School of Medicine, Indianapolis, Indiana, United States; Indiana University School of Medicine, Indianapolis, Indiana, United States; Indiana University School of Medicine, Indianapolis, Indiana, United States

## Abstract

Social connectedness has been linked to decreased rates of cognitive decline in later life. However, recent work suggests that particular social network characteristics (i.e., bonding and bridging) may buffer against age-related degeneration. The present study analyzes social network and structural MRI data of 176 older adults from the Social Networks and Alzheimer’s Disease (SNAD) study. Results indicate that increased social bridging is associated with greater grey matter (GM) volume in several limbic structures. Increased social bonding is associated with greater GM volumes in several cerebral cortex structures as well as greater volumes in some components of the limbic system. Most notably, the effects of bridging are primarily lateralized in the left hemisphere while the effects of bonding are observed mostly in the right hemisphere. These results suggest that the neurocognitive benefits of social connectedness depend on the preponderance of bridging and/or bonding ties in older adults’ social networks.

